# 纯白细胞再生障碍合并胸腺瘤、肺癌1例报告并文献复习

**DOI:** 10.3760/cma.j.cn121090-20240113-00018

**Published:** 2024-08

**Authors:** 秀丽 陈, 振杰 蔡, 榕 郑, 武强 林

**Affiliations:** 1 福建省莆田市第一医院血液内科，莆田 351100 Department of Hematology, the First Hospital of Putian City, Putian 351100, China; 2 福建医科大学临床医学部，福州 350000 School of Clinical Medicine, Fujian Medical University, Fuzhou 350000, China

## Abstract

纯白细胞再生障碍（PWCA）是一种罕见的血液系统疾病。本文报道了1例67岁的男性患者，在重度中性粒细胞减少的同时被发现合并有胸腺瘤、肺癌。结合血常规、骨髓细胞形态学、骨髓病理、流式细胞术免疫分型、胸腺病理等检查，排除纯红细胞再生障碍、骨髓增生异常综合征及其他原因后，患者诊断为胸腺瘤相关PWCA。人G-CSF持续治疗对患者PWCA无效。患者白细胞、中性粒细胞计数在环孢素治疗后有所上升，并在胸腺瘤切除术后第8天恢复正常。患者术后第40天感染新型冠状病毒时发现PWCA复发，并最终死于重症感染。因此，对于病因不明的严重中性粒细胞减少症应及时评估纵隔及骨髓情况。

## Abstract (en)

Pure white cell aplasia（PWCA）is a rare hematologic disorder. In this case study, a 67-year-old man presented with severe neutropenia along with thymoma and lung cancer. A comprehensive diagnostic approach was done which included routine blood test, bone marrow cytology, bone marrow pathology, flow cytometry, and thymic pathology. Other potential causes, such as pure red blood cell aplasia and myelodysplastic syndrome, were ruled out. The final diagnosis was determined to be thymoma-related PWCA. Continuous treatment with human granulocyte colony-stimulating factor（G-CSF）was ineffective for treating PWCA in this patient. The patient's white blood cell and neutrophil count increased following treatment with cyclosporine and subsequently returned to normal levels by the 8th day after thymectomy. A recurrence of PWCA was identified 40 days after the operation and coincided with COVID-19 infection. The patient eventually succumbed to a severe infection. Therefore, in cases of severe neutropenia with an unclear etiology, prompt evaluation of mediastinal and bone marrow status is imperative.

1983年，Levitt等[1]第一次提出了“纯白细胞再生障碍（Pure white cell aplasia，PWCA）”这个名词。PWCA是一种罕见的血液系统疾病，目前并无统一的诊断标准，其特征是外周血粒细胞缺乏症伴骨髓髓系前体细胞缺失，而红细胞、巨核细胞生成基本正常，并排除其他粒细胞减少性疾病[2]。PWCA可见于感染、服用药物、射线照射、自身免疫性疾病等人群，还有部分原因不明。我们报道1例PWCA合并有胸腺瘤、肺癌，并进行相关文献复习。

## 病例资料

患者，男，67岁，因“体检发现白细胞减少1个月余，发热1 d”于2022年10月28日入院。患者入院前1个月余在当地医院体检查血常规：WBC 1.0×10^9^/L，中性粒细胞0.3×10^9^/L，淋巴细胞0.6×10^9^/L，HGB 100 g/L，平均红细胞体积（MCV）、平均红细胞血红蛋白量（MCH）正常，PLT 199×10^9^/L。抗核抗体（ANA）谱阴性。当地医院多次予人G-CSF，白细胞恢复不明显。既往体健，无高血压、糖尿病、肝炎、恶性肿瘤等病史，近年无反复感染病史。本次入院前1 d出现发热、咽痛，体温最高达38.5 °C，伴乏力，无咳嗽、咳痰、胸闷、气短等不适，查血常规示：WBC 0.54×10^9^/L，中性粒细胞0.04×10^9^/L，淋巴细胞0.43×10^9^/L，HGB 106 g/L，MCV、MCH正常，PLT 171×10^9^/L。入院查体：体温38.5 °C，神志清楚，轻度贫血貌，咽部稍红，浅表淋巴结未触及肿大，心、肺、腹查体未见异常。实验室检查：网织红细胞计数34.6×10^9^/L，网织红细胞百分比1.00％；血免疫球蛋白IgG 8.86 g/L，IgM<0.2 g/L，IgA 1.65 g/L。尿常规、粪常规、血生化、凝血功能、血癌胚抗原、甲胎蛋白、细胞角蛋白片段19、神经元烯醇酶未见明显异常。乙型肝炎病毒核酸、人类免疫缺陷病毒抗体、梅毒螺旋体抗体、丙型肝炎病毒抗体、EB病毒核酸、新型冠状病毒核酸均阴性。胸部CT平扫（图1）示：①右肺中叶结节，性质待定，首先考虑肺恶性肿瘤（MT）；②前上纵隔结节，考虑胸腺瘤；③双肺少许慢性炎症；④双侧局限性肺气肿。肝脾、浅表淋巴结彩超检查未见异常。骨髓细胞形态学（图2A）：有核细胞增生极度减低，其中髓系原始细胞占5.0％，晚幼粒细胞占0.5％，成熟淋巴细胞占86％，形态均未见异常，中幼红细胞占1％，晚幼红细胞占3％，红细胞形态未见异常，巨核细胞全片共计12个，血小板常见。骨髓流式细胞分析：淋巴细胞约占有核细胞85.5％，比例明显增多；其中B淋巴细胞几乎不可见；原始区域细胞约占有核细胞的1.5％，分布散在；髓系细胞约占有核细胞的4.5％，比例明显降低；有核红细胞占有核细胞的7.5％。骨髓病理（图2B）：①局灶造血组织增生活跃，容量约占30％，脂肪组织增生；②粒系增生减低，少见；③造血组织内红系增生，比例偏高，以中晚幼红细胞为主，形态未见明显异常；④巨核细胞增生（0～8枚/高倍镜视野），散在分布，大小形态未见异常；⑤淋巴及浆细胞散在；⑥未见明显纤维组织增生。骨髓染色体核型分析未见异常。T淋巴细胞亚群检测（绝对计数）：总B淋巴细胞数0个/µl，总B淋巴细胞0.02％，总T淋巴细胞数500个/µl，总T淋巴细胞94.26％，辅助性T淋巴细胞数287个/µl，辅助性T淋巴细胞54.22％，抑制/细胞毒性T淋巴细胞数193个/µl，抑制/细胞毒性T淋巴细胞36.38％，NK细胞数25个/µl，NK细胞4.78％，淋巴细胞总数530个/µl，CD4/CD8比值1.49。初步诊断：①PWCA可能；②胸腺瘤；③肺MT待排；④感染性发热。患者入院后经抗感染及G-CSF升白细胞治疗，未再发热，期间监测血常规示白细胞波动在（0.43～0.65）×10^9^/L，中性粒细胞波动在（0～0.04）×10^9^/L。治疗第8天复查网织红细胞数31.2×10^9^/L，网织红细胞数百分比1.10％。治疗10 d后复查胸部CT平扫示：①右肺中叶结节，较前相仿，MT？②右肺下叶新增小结节，感染性病变？③前上纵隔结节，较前相仿，考虑胸腺瘤；④双肺少许慢性炎症；⑤双侧局限性肺气肿。患者在住院第18天（2022年11月15日）开始每天口服环孢素4 mg/kg，并转诊上海交通大学附属瑞金医院胸外科备行胸腺瘤及右肺肿物切除。患者在环孢素治疗第14天复查血常规示：WBC 1.44×10^9^/L，中性粒细胞0.27×10^9^/L，淋巴细胞0.45×10^9^/L，单核细胞0.7×10^9^/L，HGB 104 g/L，MCV、MCH正常，PLT 545×10^9^/L。患者于2022年11月30日在上海交通大学附属瑞金医院行胸腺瘤及右肺中叶结节切除术。术后右肺中叶结节病理示：右肺中叶肿瘤大小：2.0 cm×1.0 cm×0.8 cm，浸润性腺癌，中-低分化，支气管旁淋巴结未见癌转移；免疫组化：肿瘤细胞CK7（+），TTF-1（+），NapsinA（+），P40（−），SMARCA4（蛋白表达），ALK-1A4（−），Met（+），PD-L1（−），Ki-67（5％）。纵隔肿物病理形态学：小灶区肿瘤组织呈伴有淋巴样间质的微结节型胸腺瘤样形态，小灶突破包膜。免疫组化：肿瘤细胞AE1/AE3（+），CK19（+），EMA（少量+），PAX-8（−），P40（+），P63（+），CK5/6（+），CD5（背景淋巴细胞+），CD117（个别+），CD1α（背景淋巴细胞部分+），CD99（背景淋巴细胞+），CD20（少量+），CD3（背景淋巴细胞+），TdT（背景淋巴细胞+），CD34（−），BCL-2（+）, STAT-6（−/+），Langerin（−），Ki-67（背景淋巴细胞90％+）（图3）。结合形态学及免疫组化结果符合AB型胸腺瘤。患者术后第8天复查血常规示：WBC 8.41×10^9^/L，中性粒细胞5.2×10^9^/L，HGB 105 g/L，MCV、MCH正常，PLT 180×10^9^/L。患者术后未再接受环孢素治疗，未遵医嘱定期复查血常规。术后第40 天（2023年1月10日），患者因感染新型冠状病毒在当地医院住院治疗，复查血常规示：WBC 0.4×10^9^/L，中性粒细胞0×10^9^/L，淋巴细胞0.4×10^9^/L，HGB 99 g/L，MCV、MCH正常，PLT 244×10^9^/L。患者最终死于粒细胞缺乏及新型冠状病毒引起的重症感染。

**图1:**
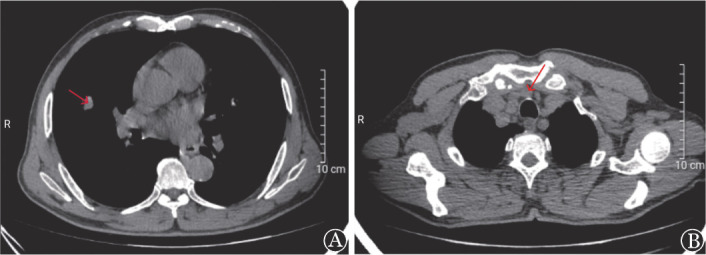
患者入院后第1次胸部CT **A** 右肺中叶结节（箭头所示）；**B** 前上纵隔结节（箭头所示）

**图2:**
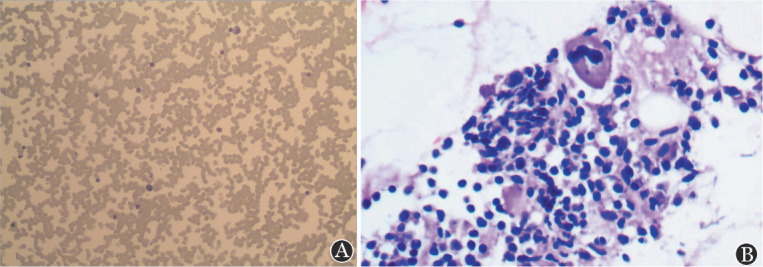
患者骨髓细胞形态学及骨髓病理 **A** 骨髓涂片见有核细胞增生极度减低（吉姆萨染色，×100）；**B** 骨髓病理见局灶造血组织增生活跃，局灶造血组织中粒系少见（苏木素-伊红染色，×400）

**图3:**
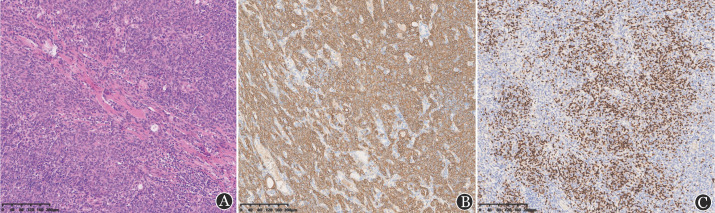
患者纵隔肿物组织形态及免疫组化结果（×100） **A** 苏木素-伊红染色见小灶区肿瘤组织呈伴有淋巴样间质的微结节型胸腺瘤样形态，小灶突破包膜；**B** CK5/6染色阳性；**C** TdT染色：背景淋巴细胞阳性

## 讨论及文献复习

胸腺在适应性免疫，特别是T淋巴细胞的成熟过程中起到了核心作用。胸腺瘤是胸腺最常见的良性肿瘤，与各种免疫介导的副肿瘤综合征相关，如重症肌无力、GOOD综合征和（或）纯红细胞再生障碍（PRCA）,但发生PWCA罕见[2]–[3]。胸腺瘤相关免疫缺陷被称为GOOD综合征，主要表现为低丙种球蛋白血症、B淋巴细胞减少或缺失、CD4^+^/CD8^+^T淋巴细胞比值倒置以及T细胞功能缺陷[4]。Kelesidis等[5]对不同文献中110例GOOD综合征患者免疫球蛋白缺乏亚型进行汇总分析后发现：74.5％的患者（82/110）为缺乏所有免疫球蛋白（IgG、IgA和IgM）的泛低丙种球蛋白血症，14.5％的患者（16/110）缺乏IgA、IgG、IgM中的两种，9.1％的患者（10/110）仅IgG水平较低，1.8％的患者（2/110）仅IgA水平较低，但并未发现单纯缺乏IgM的患者。本例患者存在胸腺瘤、B淋巴细胞缺如、低IgM丙种球蛋白血症，是否可诊断为GOOD综合征仍有待商榷。

胸腺瘤最常见的血液学副肿瘤综合征是PRCA，罕见PWCA、再生障碍性贫血（AA）、自身免疫溶血性贫血（AIHA）、获得性巨核细胞性血小板减少症（AMT）、T-细胞大颗粒淋巴细胞白血病（T-LGLL）[3],[6]–[10]。本例患者存在轻度正细胞性贫血，骨髓细胞学提示有核红细胞比例明显下降，红细胞形态未见异常，需要进一步排除PRCA的可能。PRCA是一种以正细胞正色素性贫血、网织红细胞减低和骨髓中红系前体细胞显著减低或缺如为特征的综合征，其粒系及巨核系各阶段细胞比例正常[11]。本例患者网织红细胞百分比≥1％，网织红细胞绝对值≥10×10^9^/L，并未达到PRCA诊断标准。患者骨髓细胞学虽然提示有核红细胞减少，但骨髓流式细胞术检测提示有核红细胞比例达到7.5％，骨髓病理提示造血组织内红系增生，比例偏高，以中晚幼红细胞为主，形态未见明显异常。考虑到骨髓涂片可能存在因穿刺及部位不同等原因影响有核红细胞计数，结合血常规、骨髓病理、骨髓流式免疫分型、网织红细胞等检测结果，不考虑红系造血功能障碍。本例患者骨髓细胞学可见髓系原始细胞占5％，其贫血需要进一步排除骨髓增生异常综合征（MDS）的可能。既往文献报道，G-CSF应用后5～7 d，骨髓原始细胞比例可明显增多，甚至超过20％，但对红系、巨核系没有影响，停药2周后大多数原始细胞可恢复正常[12]–[13]。本例患者在骨髓穿刺检查前已在外院接受过多次G-CSF治疗，骨髓原始细胞增多考虑与G-CSF治疗相关可能性大。遗憾的是，本例患者因经济受限，未进一步完善血液肿瘤常见基因突变二代测序技术（NGS）检测，后续也未再行骨髓穿刺检查，无法进一步验证骨髓原始细胞增多与G-CSF的相关性。同时本例患者骨髓未见病态造血现象，染色体核型未见异常，骨髓病理并不支持MDS诊断，骨髓细胞的流式细胞术检测未发现MDS相关的异常表型，并未提示红系或髓系存在单克隆细胞群，未达到MDS诊断标准[14]。本例患者外周血及骨髓中T淋巴细胞比例均明显升高，但骨髓增生极度减低，淋巴细胞绝对计数减少，CD4^+^/CD8^+^比值无升高或降低，形态学及流式细胞学均未提示异常增殖或形态异常的淋巴细胞亚群，临床表现亦无肝脾、淋巴结肿大等，因此考虑为B淋巴细胞及粒系减少引起的相对性T淋巴细胞比例增多可能性大。因经济受限，本例患者并未进行T细胞受体重排检测进一步明确T淋巴细胞的克隆性。

胸腺瘤病理类型可分为A型、AB型、B1型、B2型和B3型[15]。在PWCA相关文献报道中，A型和AB型胸腺瘤更常见[3]–[4]。在某些情况下，手术切除胸腺瘤有助于改善PWCA[2],[16]–[18]，包括本病例患者的粒细胞缺乏在胸腺瘤切除术后也得到了短暂的完全恢复，表明胸腺瘤与PWCA相关。我们报道的这例患者在发现胸腺瘤的同时发现浸润性肺腺癌，但目前无文献支持肺肿瘤与胸腺瘤、PWCA之间存在相关性，考虑为合并症。胸腺瘤相关PWCA的病因目前暂不明确，但胸腺是T细胞发育、成熟的重要器官，也是自身反应性T细胞发生阴性选择的地方[19]，因此多数认为PWCA与自身免疫相关。目前多数胸腺瘤相关PWCA病例表现出B淋巴细胞明显减少或确如，并可观察到这些患者血清对粒细胞和巨噬细胞集落形成单位的生长抑制[1],[17],而体外自体和异基因造血干细胞的生长也受到患者血清（特别是IgG部分）的抑制[20],表明患者血清中存在抑制因子。与胸腺瘤相关的自身免疫最可能的解释是，胸腺内肿瘤生长导致的损伤降低了其维持自我耐受的能力，并为自身免疫性疾病的发生提供了契机[21]。而胸腺瘤患者自我耐受性丧失的可能机制有：①不成熟的肿瘤性T细胞会使自身反应性淋巴细胞逃逸；②HLA-DR表达降低等肿瘤性遗传改变促进自身免疫的出现；③体液免疫与细胞免疫失调，导致自身反应性T细胞激活B细胞产生自身抗体[4],[21]。

目前对于胸腺瘤相关PWCA的治疗并没有统一的标准，治疗的基础是假设粒细胞缺乏症是由自身免疫所介导的。G-CSF通常对粒细胞计数影响较小[4]。但也有学者对10例胸腺瘤伴粒细胞缺乏病例进行总结后发现存在两种骨髓功能障碍的模式：一种为早幼粒细胞成熟停滞，其中1例早幼粒细胞成熟停滞的患者，对G-CSF治疗有效，但需长期维持治疗；而另一种为骨髓粒系生成功能完全缺失[22]。目前未发现其他病例报道提示单纯G-CSF治疗对粒细胞计数有影响。本病例患者在G-CSF连续治疗10 d后，粒细胞计数依然没有明显升高。

胸腺瘤切除术理论上可以通过切除为自身反应性T细胞提供抗原刺激的肿瘤组织来解决自身免疫表现。但目前报道的多数病例以及本文报道的病例中，单纯切除胸腺瘤并不足以解决问题。部分PWCA患者包括本例患者在胸腺瘤切除术后出现了短暂的粒细胞升高，但很快再次出现粒细胞缺乏[2],[4],[16]–[18]，甚至曾有病例在胸腺瘤切除术后才首次出现PWCA表现[3]。可见胸腺切除术需要联合其他治疗。鉴于胸腺瘤相关PWCA与自身免疫可能存在相关性，免疫抑制剂也许是个不错的选择。

免疫抑制剂环孢素对T细胞具有免疫抑制作用，在治疗胸腺瘤相关PWCA中取得了良好的疗效[2]–[4],[16]–[17]。环孢素作为一种安全性已知、有广泛使用经验和可实现浓度监测的药物，是现有文献支持的最成功的胸腺瘤相关PWCA治疗药物。环孢素治疗的目标谷浓度为200～400 ng/ml，粒细胞恢复一般在7～10 d内发生，通常采用维持性治疗，在4～6个月后逐渐减量，直至完全停止[2]–[4]。我们报道的这例患者在环孢素治疗2周后，粒细胞缺乏有所改善，并在胸腺瘤切除术后，白细胞及中性粒细胞计数均恢复到正常，未再继续口服环孢素治疗。本例患者再次出现中性粒细胞明显下降时伴随有新型冠状病毒感染。虽然新型冠状病毒感染也可导致白细胞减少，但多以淋巴细胞减少为特征，中性粒细胞减少并不常见，粒细胞缺乏更罕见[23]–[24]。因此考虑患者再次出现粒细胞缺乏与新冠病毒感染造成免疫功能紊乱，PWCA复发相关可能性更大。免疫抑制剂阿伦单抗（Alemtuzumab）是一种针对T、B淋巴细胞和单核细胞上存在的CD52抗原的人源化单克隆抗体，已经成功应用于自身免疫性骨髓衰竭[4]。两例PWCA患者在使用阿伦单抗后的第1个月内达到了完全缓解[25]。另1例胸腺瘤合并PWCA患者，在G-CSF和血浆置换治疗失败后，使用阿伦单抗在12 d内实现了粒细胞恢复；然而，患者粒细胞缺乏在治疗5个月后出现复发，但复发后再次使用阿伦单抗仍然有效[26]。另外，静脉注射免疫球蛋白[2]、血浆置换术[20]、霉酚酸酯[4]也在部分病例中对PWCA有一定的疗效，而激素与环磷酰胺则被认为对粒细胞缺乏无效[20]。

综上，本例患者在排除PRCA、MDS及其他原因后，可诊断胸腺瘤相关PWCA。今后对于病因不明的严重中性粒细胞减少症应及时评估纵隔及骨髓情况。对胸腺瘤合并PWCA的患者，可以尝试手术联合免疫抑制剂的治疗方案。鉴于PWCA复发的风险很高，有必要对胸腺瘤患者进行长期随访，监测血常规及免疫功能。
